# A Multi-Biomarker Approach in European Sea Bass Exposed to Dynamic Temperature Changes under Dietary Supplementation with *Origanum vulgare* Essential Oil

**DOI:** 10.3390/ani11040982

**Published:** 2021-04-01

**Authors:** Francesca Rita Dinardo, Aristide Maggiolino, Elisabetta Casalino, Michele Deflorio, Gerardo Centoducati

**Affiliations:** Department of Veterinary Medicine, University of Bari Aldo Moro, Casamassima km 3, 70010 Valenzano, Italy; francesca.dinardo@uniba.it (F.R.D.); aristide.maggiolino@uniba.it (A.M.); michele.deflorio@uniba.it (M.D.); gerardo.centoducati@uniba.it (G.C.)

**Keywords:** oxidative stress, biochemical parameters, oregano essential oil, temperature, sea bass

## Abstract

**Simple Summary:**

Temperature fluctuations may induce metabolic and physiological imbalances over marine organisms, involving reproduction, growth, immune response, osmoregulatory capacity, and antioxidant defenses. It is of great importance to find tools, including nutritional interventions on farms, able to reduce such imbalances and the consequent stress for animals. In light of this perspective, we investigated the correlations between temperature and metabolic performance in sea bass fed on diet containing oregano essential oil. Under the condition of our study, thermal changes affected the levels of several biomarkers (e.g., triglycerides and cholesterol) highlighting an attempt to provide for additional energy, to counterbalance the oxidative damage, and to maintain cell homeostasis. On the other side, the activity of antioxidant enzymes, TBARS levels, and the energetic balance seemed to benefit from the intake of oregano essential oil under exposure to thermal changes.

**Abstract:**

A feeding trial for 150 days was carried out to evaluate the cross-effects between oregano essential oil (EO) dietary supplementation and dynamic temperature change in sea bass. Under exposure to rising temperature (13–25 °C), fish were fed with a control diet (CD) and two experimental diets supplemented with 100 (D100) and 200 ppm (D200) of EO. Feed inclusion of EO promoted the activity of antioxidant enzymes in sea bass exposed to increasing temperature. Consistently with the temperature rise, TBARS concentrations increased in CD and D200 groups, whereas were almost stable in D100. Trend of blood glucose in fish fed on CD was likely affected by glycogenolysis and gluconeogenesis. Similarly, the depletion of triglycerides and cholesterol in fish fed on CD likely supported the energy cost of gluconeogenesis. On the other hand, the reduction of glucose, triglycerides, and cholesterol in D100 and D200 was mainly attributable to the hypoglycemic and hypolipidemic effects of EO. The higher levels of serum protein observed in D100 and D200 groups were also associated to a reduced thermal stress compared to CD. EO dietary supplementation may be a promising strategy to alleviate the negative effects of temperature shift on sea bass physiological and oxidative state.

## 1. Introduction

Anthropogenic impacts, including the emission of carbon dioxide, the overuse of pesticides and the discharge of industrial and household chemical wastes have been considered the main causes of environmental temperature global change. Most aquatic communities are particularly vulnerable to the thermal fluctuations, which may affect ecological aspects and anatomical (e.g., body insulation and locomotor system) and physiological functions of aquatic organisms (e.g., blood–water countercurrent respiratory system, metabolic rate, and osmoregulation) [1,2,3]. Several studies have previously described the strong relationship between metabolic processes and temperature, whose variations may slow down or accelerate enzyme-catalyzed reactions [4,5]. Low and high temperature extremes decrease the secretion and activity of digestive enzymes, leading to minor feed intake and growth efficiency. Alternatively, temperatures close to the optimum level promote food digestion by increasing the metabolic rate or reducing the intestinal transit time [6]. Continuous exposure to thermic variations may counter sex ratios and reproduction status, making fish sterile or sexually incompetent [7]. Temperature is the major driver of embryogenesis and gonadal differentiation processes [8,9]. For these reasons, the environmental temperature has been defined as the main abiotic regulatory factor of basic physiological processes involved in animal growth, reproduction, and welfare [10]. In order to adapt its metabolic rate to thermal variations, aquatic fauna has developed a temperature range with maximum and minimum tolerance limits. Physiological alterations impair the cellular redox balance with the consequent formation of reactive oxygen species (ROS), which may damage vital biomolecules such as DNA, protein, and lipid [11]. For these reasons, antioxidant enzymes (e.g., superoxide dismutase and catalase) are used as biomarkers of fish health status due to their pivotal role in cellular defense systems against temperature-induced oxidative stress [12,13].

Plant-enriched diets may represent an effective strategy to increase fish immunity and disease resistance in aquaculture. Several studies have revealed the immunostimulant, antioxidant, and antimicrobial potential of plant-based supplementation in fish diet [14,15,16]. The use of plant supplements can also reduce the mortality rates and improve growth and feed assimilation, contributing to a better optimization of aquaculture resources. Whether medicinal plants can be administered to fish in several ways (e.g., intramuscular and intraperitoneal injection, immersion, or baths), oral administration seems to be the most suitable for aquaculture. Plants can be administered as a whole plant or parts (leaf, root, seed, fruit) and can either be used fresh or as powder, plant-extract, or essential oils (EO) [17,18]. Recently, much attention has been given to EO-enriched diets in order to preserve the fish healthiness and to minimize the farming cost, as well as to improve the fillet nutritional quality [16]. Blood parameters act as insightful markers of physiological perturbations depending on extrinsic (e.g., temperature, season, dissolved oxygen, water quality, stocking density, photoperiod) and intrinsic factors (e.g., age, nutritional state, species) [19]. Thus, previous studies investigated the levels of several blood parameters after EO administration, showing an enhanced health status of treated fish [20,21,22]. Carvacrol is the main component of *Origanum vulgare* (L.) EO and has a broad spectrum of biological activity [23]. Consequently, carvacrol-rich oregano oils have been applied in farmed fish diet as growth- and health-promoter. Anyway, the specific mechanisms behind the observed physiological effects of *Origanum vulgare* (L.) EO or carvacrol are still poorly described, as well as more research is needed to establish the most suitable preparations and the most appropriate treatment strategies. Adequate dosing is crucial to obtain the desired effects, since most of the plant bioactive molecules may result toxic or antinutritional at high levels. Treatment duration is another important parameter, since it directly affects the treatment effectiveness [17,18].

Due to its wide thermal tolerance range, large acclimation capacity, and low generation times, sea bass (*Dicentrarchus labrax* L.) has acquired a considerable commercial importance [5]. Anyway, the climate change effects are predicted to represent great ecological challenges for this species [24,25]. Sea bass grows best at temperature ranging from 22 to 24 °C, depending on the geographic region. In the wild, sea bass moves between deeper and shallow water, and seasonally between the open sea and lagoons/estuaries. Farmed sea bass is cultured in cage systems along the coast; therefore, it cannot alter their position to cope with daily and/or seasonally temperature changes and it has to face with continuous metabolic adjustments [26]. European sea bass may represent a good biological model for evaluating the impacts of temperature fluctuations in aquatic ecosystem [27]. In our study, we tested the effects of oregano EO-enriched diets on the physiological and oxidative state of sea bass exposed to temperature shifts. Aiming to provide a scientific base for effective aquaculture practices, we collected a broad range of fish responses under different doses of EO and during a long treatment period.

## 2. Materials and Methods

### 2.1. Animals and Experimental Design

European sea bass (*Dicentrarchus labrax* (Linnaeus, 1758)) were obtained from the commercial fish farm ‘‘Ittica Caldoli’’ (San Nazario, Lesina, FG, Italy). All fish were visually healthy and showed no clinical signs of abnormalities or infestation. Prior to the trial, 420 fish (12.48 ± 0.7 g body weight and 15.0 ± 0.90 cm total body length) were acclimated to 13 °C for 15 days. During this period, they were fed twice a day until apparent satiation with a commercial food pellet provided by Veronesi Mangimi A.I.A. S.p.A (Verona, Italy). The nutritional composition of commercial feed is reported in Table 1.

**Figure 1:**
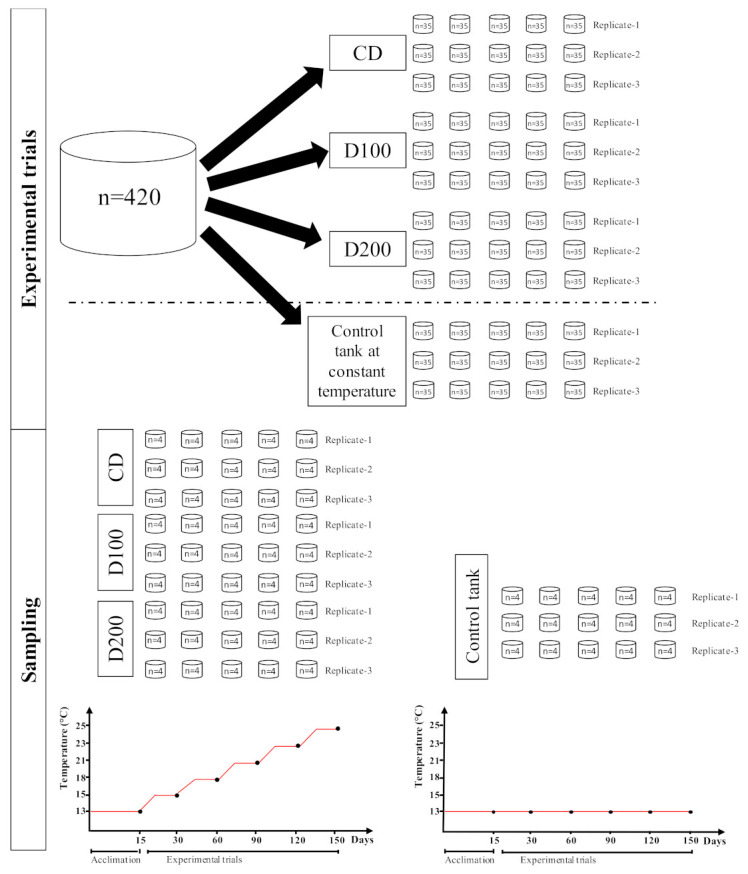
Schematic design of experimental trials and sampling protocol.

Then, specimens were randomly distributed in 12 cylindrical fiberglass 2000-L tanks (4 treatments × 3 replications; *n* = 35 individuals per tank). Nine tanks were exposed to same temperature conditions because they were inter-connected and linked to a re-circulating system, with a water flow of 7200 L/h (about eight total volume renewal per day) and equipped with mechanical and UV filters, a skimmer, a 3000 L biological filter and a 3000 L/h heat/cool pump. Ten percent of the water volume was renewed with reconstituted water every week. These tanks were placed in an air-conditioned room to support the increase in water temperature and to avoid heat loss during the experimental trial. There was constant aeration of the water with supplemental oxygen to keep dissolved oxygen values within the optimal range. Fish were progressively exposed to five experimental temperatures: 15, 18, 21, 23, and 25 °C, which reflected the natural water temperature range occurring in the southern Mediterranean region from mid-winter to mid-summer (https://www.seatemperature.org, accessed on 4 November 2019) (Figure 1). The water temperature was monthly increased regularly (2–3 °C month^−1^) according to the seasonal trend. Four days before starting each exposure phase, water temperatures were progressively increased by 0.50–0.75 °C. After this period, the required experimental temperatures were kept constant for the remaining 26 days. The other three tanks were used as control at constant temperature (data not shown). These ones were placed in a different air-conditioned room and water temperature was constantly kept through a heat/cool pump at 13 °C during all the experimental trial (Figure 1). The tanks were linked to re-circulating system with a water flow of 2400 L/h (about eight total volume renewals per day) and equipped with mechanical and UV filters, a skimmer, a 1000 L biological filter and a 1000 L/h heat/cool pump. In control tanks, constant aeration of the water was sufficient to maintain optimal dissolved oxygen values. All tanks were maintained at a 14:10 L: D light–dark regime. During the experimental trial, the water quality parameters (e.g., temperature, dissolved oxygen, pH, total ammonia, nitrite, and nitrate) were monitored daily. Data about water temperature, dissolved oxygen, pH and salinity were collected by means of a tester HI-9829 (Hanna Instruments, Padova, Italy) whereas total ammonia, nitrite, and nitrate were measured with colorimetric kit (Testlab Marin, JBL). The mean temperature values during each exposure phase were 13.05 ± 0.03 °C; 15.35 ± 0.01 °C; 17.90 ± 0.08 °C; 21.20 ± 0.01 °C; 23.15 ± 0.03; and 25.07 ± 0.21 °C. These range values were marked as groups 13, 15, 18, 21, 23, and 25 °C. During the 150-days experimental period, specimens were maintained under the following conditions: 7.4 ± 0.5 mg/l of dissolved oxygen, 7.5 ± 0.1 of pH and 30 ‰ ± 2 of salinity. Ammonia (NH_4_^+^), nitrite (NO_2_^−^), and nitrate (NO_3_^−^) concentrations were kept below 0.05 mg/L, 0.20 mg/L, and 2.0 mg/L, respectively.

### 2.2. Experimental Diets

Fish were fed on three experimental diets: a control diet (Basic 3 commercial food pellet) and two diets which were supplemented with different concentrations of oregano (*Origanum vulgare* L., 1753) essential oil (EO). The EO used in this study was obtained from Farmalabor S.r.l. (Canosa di Puglia, Italy). The chemical composition provided by the manufacturer is reported in Table 2. Supplemented diets were prepared according to the protocol described by Dairiki et al. [28] and Dinardo et al. [29]. Briefly, oregano EO was dissolved in grain alcohol to prepare EO suspensions at different concentrations. Commercial pellets were ground and the resultant powder was mixed with the EO suspensions to reach the final concentrations of 100 (D100) and 200 ppm (D200) of EO [30]. In the control diet (CD), the same amount of pure grain alcohol was added to the feed, without EO supplementation. The mixtures were homogenized, pelleted, left to dry for 24 h at 25 °C, and stored at −18 °C until feeding. Each diet was tested in triplicate (three tanks per treatment). The fish were fed twice a day for 150 days until apparent satiety.

Animal management and sampling was carried out aiming at minimizing stress and health risks. The experiments were performed in accordance with the Italian guidelines for animal care (DL 26/14) and the European Communities Council Directive (2010/63/UE), and approved by the General Directorate of Animal Health and Veterinary Drugs of Ministry of Health, with authorization no. 444/2019-PR on 12 June 2019.

### 2.3. Blood Sampling

Every 30 days, twelve fish from each treatment (4 fish per tank) were randomly sampled, anesthetized with fish clove oil at a dose of 30 mg/L and soaked in ice-slurry to achieve death by hypothermia [31,32]. Blood samples (ca. 1 mL) were drawn from the caudal vein, using a 1-mL syringe, collected in plastic tubes and allowed to clot at room temperature. Subsequently, serum was separated by centrifugation at 3000 rpm for 5 min, stored at −80 °C and analyzed one week later.

### 2.4. Fish Performance

During and at the end of the feeding trial, the fish were weighed (g/fish) and the specific growth rate (SGR) was calculated as follow: SGR = 100 * [Ln (final body weight)—Ln (initial body weight)]/days of feeding trial.

### 2.5. Oxidative Stress Parameters

The thiobarbituric acid-reactive substances (TBARS) assay was performed in serum to quantify the peroxidative damage to lipids that occurs with free radical generation [33]. Free radical damage to lipids result in the production of malonaldehyde (MDA), which reacts with thiobarbituric acid (TBA) under conditions of high temperature and acidity generating a chromogen that can be measured spectrophotometrically at 535 nm. TBARS levels were reported as nmol MDA/mL.

Serum superoxide dismutase (SOD, EC1.15.1.1) activity was carried out as described by Misra [34]. The enzymatic activity was based on the 50% inhibition rate of epinephrine auto-oxidation at 480 nm. SOD activity was expressed as U/mL. Serum catalase (CAT, EC 1.11.1.6) activity was evaluated by following the decrease in absorbance of H_2_O_2_ at 240 nm [35]. One unit of enzyme activity was defined as the amount of enzyme required to degrade 1 μmol of H_2_O_2_ in 60 s. CAT activity was expressed as U/mL. Each sample analysis was performed in triplicate.

### 2.6. Serum Biochemical Analysis

Bradford assay [36] was carried out to quantify the total protein levels in each sample, using bovine serum albumin as standard. The protein concentrations were expressed as g/dL. Cholesterol, triglycerides, and glucose were measured using commercial colorimetric kits following manufacturer instructions (FAR S.r.l., Pescantina, VR, Italy), and concentrations were reported as mg/dL. Each sample analysis was performed in triplicate.

### 2.7. Statistical Analysis

Treatments were performed in triplicate. Results were reported as means ± standard deviations. Fish were used as statistical units (*n* = 12) after verifying the absence of a tank effect through a three-way nested analysis of variance (ANOVA), with temperature and feeding treatment as fixed factors and the tank as aleatory factor. Growth performances data were submitted to one-way ANOVA. A two-way ANOVA was used to analyze the effect of temperature and feeding treatments on oxidative stress biomarkers and biochemical parameters. ANOVA analyses were followed by the Tukey post hoc tests with significance level of 5%. In addition, oxidative stress biomarkers and biochemical parameters were subjected to principal components analysis (PCA) and statistical differences were evaluated using two-way PERMANOVA analyses. PERMANOVA test was performed with 999 permutations, with Euclidean distances as the distance measure and obtaining *p*-values from permutations. Data were analyzed using Statistica 13.0 (Statsoft Inc., Tulsa, OK, USA) and PAST 4.05 (University of Oslo, Oslo, Norway).

## 3. Results

### 3.1. Growth Parameters

After 150 days, fish fed on diet containing 100 ppm (D100) oregano EO showed a significantly (*p* < 0.05) higher final body weight compared both to control (CD) and 200 ppm EO diet (D200) (Table 3 and Supplementary Table S1). Similarly, the highest (*p* < 0.05) specific growth rate (SGR) value was found in D100. Weight values recorded at each sampling point were shown in Table S2 of the Supplementary Files.

### 3.2. Oxidative Stress Biomarkers

**Figure 2:**
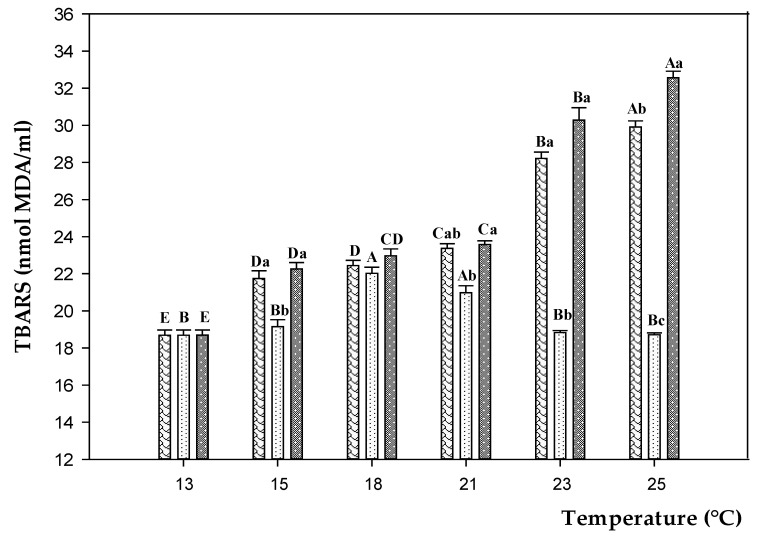
TBARS levels in sea bass fed on control diet (CD, 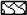) and experimental diets with different concentration of oregano essential oil: 100 ppm (D100, 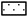) and 200 ppm (D200, 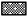) and, exposed to different water temperature (13, 15, 18, 21, 23, and 25 °C). Data are reported as means ± standard deviations (at each temperature value, *n* = 12 per treatment). Different capital letters (A–E) indicate significant differences (*p* < 0.05) among water temperature within the same diet. Different lowercase letters (a–c) indicate significant differences (*p* < 0.05) among diets within the same water temperature.

According to the temperature rise from 13 to 25 °C, TBARS concentrations increased significantly in CD and D200 groups (plus 60 and 74%, respectively) (Figure 2 and Supplementary Table S1). Excepted at 25 °C, no significant differences (*p* > 0.05) were observed between CD and D200 during the experimental trials. Conversely, in fish fed on D100, TBARS levels (*p* < 0.05) were stable between 13 and 15 °C, then increased rising the temperature from 15 to 21 °C (plus 10%). At 23 and 25 °C TBARS levels in D100 dropped again to the values found at 13 and 15 °C.

**Figure 3:**
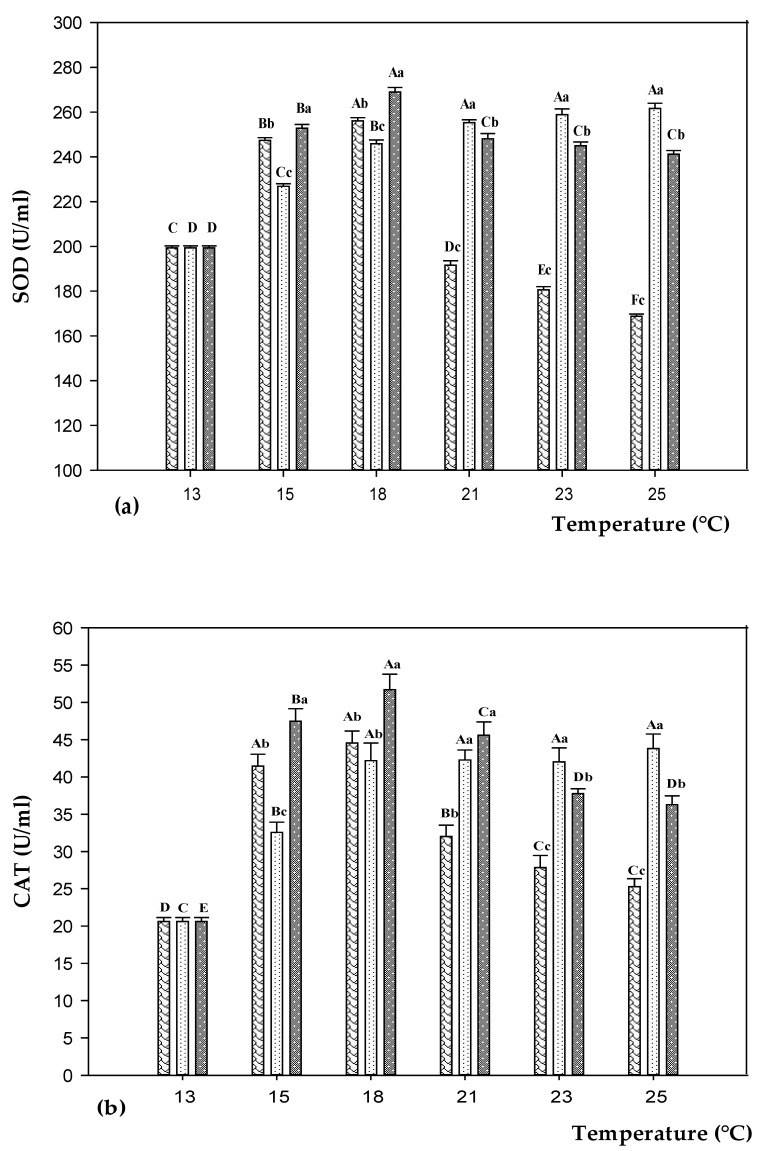
SOD (**a**) and CAT (**b**) levels in sea bass fed on control diet (CD, 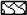) and experimental diets with different concentration of oregano essential oil: 100 ppm (D100, 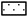) and 200 ppm (D200, 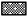) and, exposed to different water temperature (13, 15, 18, 21, 23, and 25 °C). Data are reported as means ± standard deviations (at each temperature value, *n* = 12 per treatment). Different capital letters (A–F) indicate significant differences (*p* < 0.05) among water temperature within the same diet. Different lowercase letters (a–c) indicate significant differences (*p* < 0.05) among diets within the same water temperature.

Superoxide dismutase (SOD) and catalase (CAT) activities were significantly (*p* < 0.05) affected by temperature and feeding treatments (Figure 3 and Supplementary Table S1). SOD activity increased significantly (*p* < 0.05) in both control and experimental groups rising the temperature from 13 to 18 °C (plus 23–35%), with the highest (*p* < 0.05) levels observed in fish fed on D200 diet (Figure 3a). Switching from 18 to 25 °C, enzyme levels showed different trends according to diet. SOD activity significantly (*p* < 0.05) dropped in fish fed on CD (minus 34%), whereas it remained almost stable in D100 groups. A slight but significant (*p* < 0.05) decrease of SOD activity was observed in D200 groups switching from 18 to 25 °C (minus 10%). The values of CAT activities were consistent with SOD activities trend (Figure 3b). Switching from 13 to 18 °C, the activity levels increased significantly (*p* < 0.05) in fish fed on CD and D200 diets (plus 116% and 151%, respectively), and then continually decreased (minus 43% and 30%, respectively). Overall, D200 groups showed higher (*p* < 0.05) activity than CD. In fish fed on D100, CAT activity increased (*p* < 0.05) at 15 °C and 18 °C, and then remained almost stable during the experimental trials.

### 3.3. Serum Biochemical Parameters

**Figure 4:**
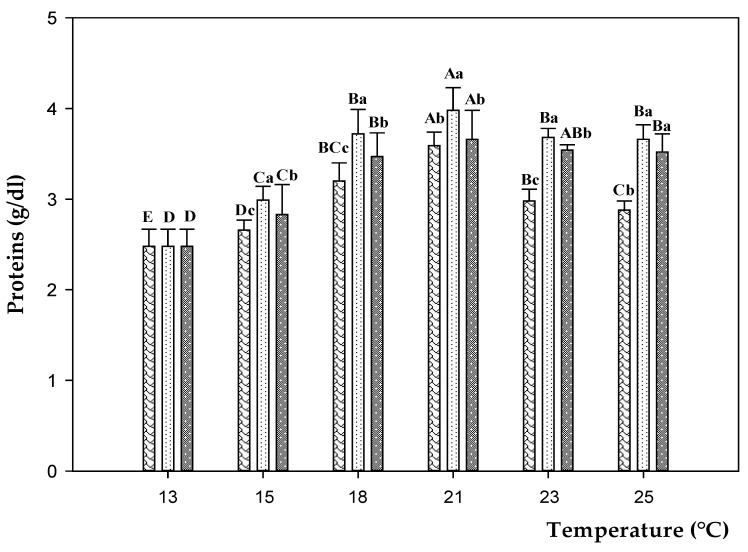
Protein levels in sea bass fed on control diet (CD, 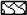) and experimental diets with different concentration of oregano essential oil: 100 ppm (D100, 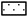) and 200 ppm (D200, 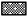) and, exposed to different water temperature (13, 15, 18, 21, 23, and 25 °C). Data are reported as means ± standard deviations (at each temperature value, *n* = 12 per treatment). Different capital letters (A–E) indicate significant differences (*p* < 0.05) among water temperature within the same diet. Different lowercase letters (a–c) indicate significant differences (*p* < 0.05) among diets within the same water temperature.

The total protein concentration fluctuated during the experimental trials (Figure 4 and Supplementary Table S1). According to the temperature rise, the protein levels followed an increasing (*p* < 0.05) trend until 21 °C (plus 45–60%) under all experimental conditions. Compared to CD, higher (*p* < 0.05) values were found at 21 °C in fish fed on D200 diet and, especially, D100. Temperature shifts from 21 to 25 °C caused a significant decrease (*p* < 0.05) of total protein in fish fed on CD (minus 20%), and to a lesser extent D100 and D200 groups (minus 8 and 4%, respectively). No significant differences (*p* > 0.05) were observed for the protein levels between fish fed on 100 and 200 ppm diets at 25 °C.

**Figure 5:**
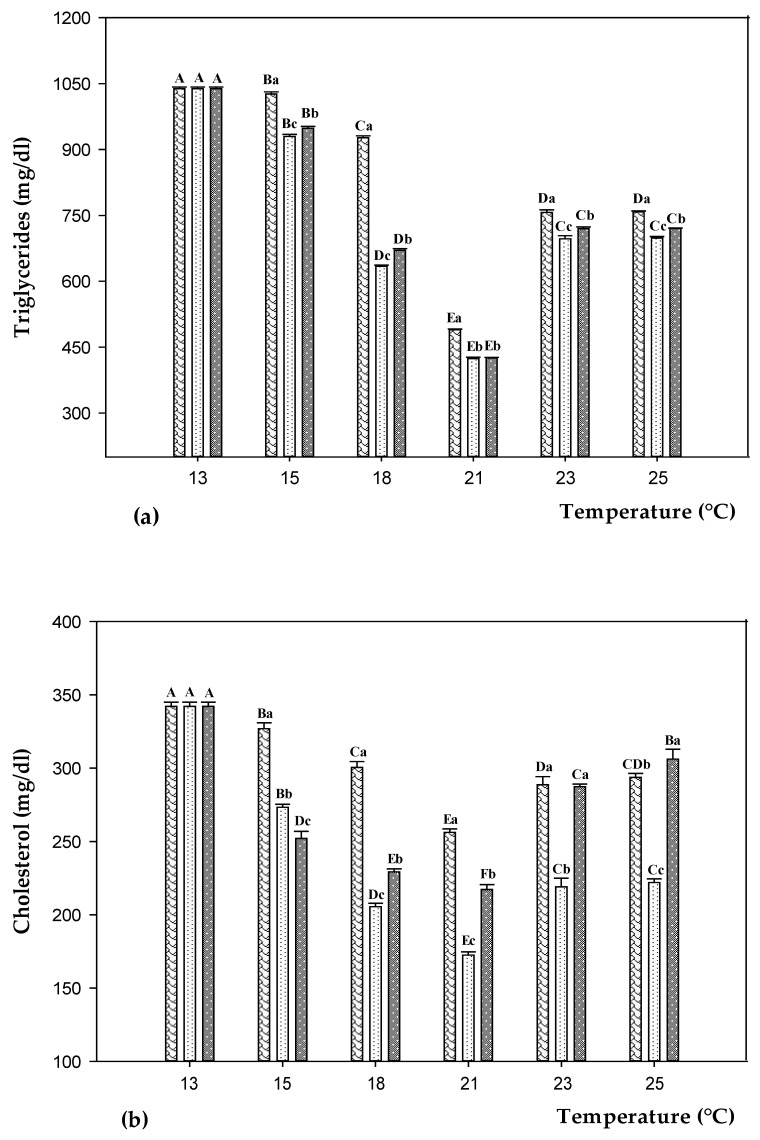
Triglycerides (**a**) and cholesterol (**b**) levels in sea bass fed on control diet (CD, 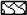) and experimental diets with different concentration of oregano essential oil: 100 ppm (D100, 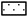) and 200 ppm (D200, 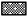) and, exposed to different water temperature (13, 15, 18, 21, 23, and 25 °C). Data are reported as means ± standard deviations (at each temperature value, n = 12 per treatment). Different capital letters (A–F) indicate significant differences (*p* < 0.05) among water temperature within the same diet. Different lowercase letters (a–c) indicate significant differences (*p* < 0.05) among diets within the same water temperature.

Serum triglycerides levels also showed a temperature-related trend (Figure 5a and Supplementary Table S1). The triglycerides content decreased switching from 13 to 21 °C (minus 53–59%) under all experimental conditions. In particular, a dramatic drop was observed between 15 and 18 °C in D100 and D200 groups (minus 32 and 29%, respectively). Switching from 21 to 23 °C, the trend was inverted (plus 55–69%). No changes (*p* > 0.05) were observed between 23 and 25 °C. Overall, the lowest (*p* < 0.05) triglycerides levels were always found in D100 and D200 groups. A similar pattern was observed for cholesterol levels (Figure 5b and Supplementary Table S1). Switching from 13 to 21 °C, the cholesterol content constantly went down (minus 25–50%) (*p* < 0.05) under all experimental conditions. Within the range 15–21 °C, the lowest (*p* < 0.05) values were recorded in fish fed on both D100 and D200. At 23 °C the cholesterol levels significantly (*p* < 0.05) increased in all groups (plus 13–32%) with respect to 21 °C. Switching from 23 to 25 °C a slight increase (*p* < 0.05) was found (plus 7%) only in fish fed on D200, whereas no changes (*p* > 0.05) were observed in CD and D100. Within the range 23–25 °C, the lowest (*p* < 0.05) values were recorded in fish fed on D100.

**Figure 6:**
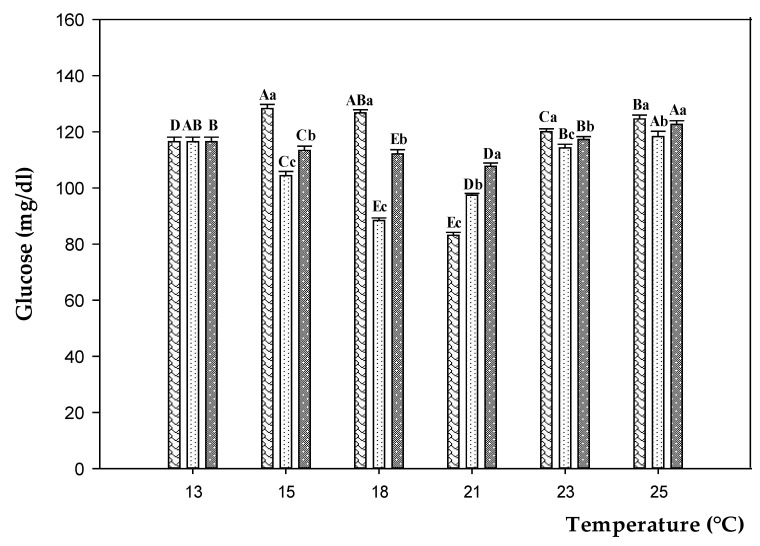
Glucose levels in sea bass fed on control diet (CD, 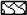) and experimental diets with different concentration of oregano essential oil: 100 ppm (D100, 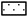) and 200 ppm (D200, 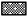) and, exposed to different water temperature (13, 15, 18, 21, 23, and 25 °C). Data are reported as means ± standard deviations (at each temperature value, *n* = 12 per treatment). Different capital letters (A–E) indicate significant differences (*p* < 0.05) among water temperature within the same diet. Different lowercase letters (a–c) indicate significant differences (*p* < 0.05) among diets within the same water temperature.

During the experimental trials, the glucose levels were affected both by temperature and feeding treatments. The glucose content in fish fed on CD increased switching from 13 to 18 °C (plus 9%), significantly decreased at 21 °C (minus 34%), and increased (*p* < 0.05) again with the temperature rise to 25 °C (plus 50%) (Figure 6 and Supplementary Table S1). Conversely, in D100 and D200 the glucose concentration significantly went down (*p* < 0.05) switching from 13 to 18 °C (minus 24 and 12%, respectively), and increased (*p* < 0.05) again rising the temperature to 25 °C (plus 34 and 20%, respectively). The lowest glucose levels were observed in D100 groups, excepted at 21 °C, when the lowest level was in fish fed on CD.

### 3.4. Multivariate Analysis

**Figure 7:**
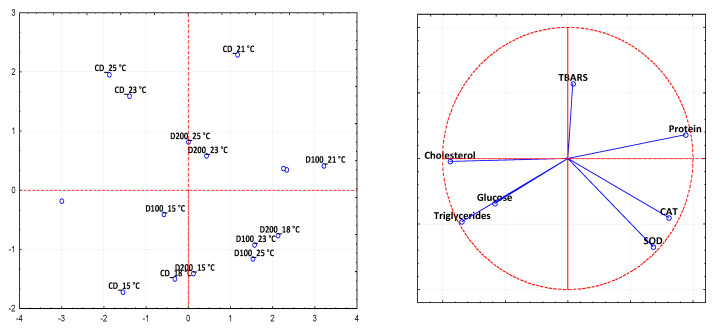
Score (**a**) and loading (**b**) plots of first and second principal components after principal component analysis performed on oxidative stress biomarkers and serum biochemical parameters in sea bass fed on control diet (CD) and experimental diets with different concentration of oregano essential oil (100 ppm (D100) and 200 ppm (D200)) and, exposed to different water temperatures (13, 15, 18, 21, 23, and 25 °C).

The principal component analysis (PCA) biplot was applied to oxidative stress biomarkers and biochemical parameters (Figure 7). Permanova analyses indicated the significance of temperature (*p* = 0. 001), diet (*p* = 0. 001) and their interaction (*p* = 0.001), giving importance to PCA analysis. The two components (factor 1 and factor 2) explained ca. 76% of total variance. PCA analysis showed that control and experimental groups exposed at low temperatures (13, 15, and 18 °C) were well separated on the plane from the same groups exposed to high temperatures (21, 23, and 25 °C), with some exceptions. Fish fed on CD and D200 diets and exposed to 21–25 °C were scattered on the upper part of the plane and shared the highest TBARS levels. On the contrary, D100 groups exposed to the same temperature were scattered on the right zone of the plane, and were mainly distinguished by the improved antioxidant enzyme activity and high serum protein levels.

## 4. Discussion

Temperature fluctuations may induce metabolic and physiological imbalances over marine organisms. Aiming to find nutritional interventions on farms, able to reduce such imbalances, we investigated the correlations between temperature and metabolic performances in sea bass fed on oregano EO. In the present study, apart from the water temperature shifts, the other environmental conditions were held constant, including photoperiod and water quality [9].

It is known that one of the first signs of stress in fish undergoing temperature changes is the alteration of the redox state [37]. TBARS are good indicators of induced oxidative damage in cells. By raising temperature, our results showed an increased serum level of TBARS in fish fed on CD, especially switching from 21 to 25 °C when the activity of antioxidant enzymes (SOD and CAT) dropped. Results are in line with previous studies describing the oxidative stress in sea bass due to stressful temperatures [38]. This was likely due to the inability of the antioxidant enzyme machinery to compensate for ROS-generating stressful conditions [39,40]. Feed inclusion of EO reduced TBARS levels and promoted the activity of antioxidant enzymes. In particular, the protective effect against oxidative damages occurred with supplementation of 100 ppm diet, whereas it was negligible with 200 ppm. The interactive effect of EO and stressful temperatures has not been studied before and makes it difficult to compare our findings with others. High doses of EO were previously reported to be inefficacy or deleterious in sea bass dietary supplementation by Dinardo et al. [29]. On the other hand, high temperatures may also result in higher toxicity of chemicals, by affecting the uptake and detoxification mechanisms, the metabolic rates, and the enzymatic activities [24,41]. Dietary supplementation with 100 ppm EO boosted the antioxidative status of sea bass through a considerable elevation of serum SOD and CAT. The same effect was not observed with D200, especially under the warmer temperature, likely due to the inability of the antioxidant enzyme machinery to compensate for the presence of both stressors (high temperature and high EO dose exposure). This resulted in a diminished protective action against oxidative stress and, ultimately, an increased lipid peroxidation [39].

The antioxidant properties of EO have been widely established, and attributed to the presence of phenols such as carvacrol, having a hydroxyl group in the phenolic ring lending a radical scavenging or metal chelating activity [42,43]. Within this frame, we speculated that the antioxidant constituents of EO counteracted the oxidative stress induced by temperature increase. We also hypothesized a beneficial effect of EO going beyond the inherent antioxidant activity of carvacrol. As previously reported, the antioxidant enzyme machinery can be impaired when excessive oxidative damage occurs and substrate is accumulated (negative feedback) [39,40]. Supporting our thesis, carvacrol administration was previously shown to recover the activities of CAT and SOD and to mitigate the lipid peroxidation in mice [44].

Serum glucose level is another index of thermal stress, and supply of glucose in bloodstream allows to cope with high metabolic needs in stressed organisms [45,46]. In the present report, fish fed on CD showed an increase of serum glucose content upon exposure to 15 and 18 °C. As primary response to cold stress, the stimulation of glycogenolysis by catecholamines promotes the breakdown of hepatic glycogen and the release of glucose into the blood [47,48]. The same hyperglycemic responses were reported in many species, such as sea bream, Nile tilapia, silver catfish, milkfish, and grass carp [27,49,50,51]. Trend of blood glucose in control group at temperatures between 21 and 25 °C could be a consequence of depletion of hepatic glycogen stores and the subsequent activation of gluconeogenesis. The stimulation of glycogenolysis and gluconeogenesis in sea bass subjected to thermal stress has been previously reported in several studies [9,38,52]. Islam et al. [38] detected low amount of blood glucose in fish reared both at low and high temperature extremes (8 and 32 °C). Samaras et al. [9] found lower levels of circulating glucose in sea bass exposed to a temperature of 15 °C with respect to 25 °C. A direct comparison of results from different studies is not always possible due to differences in experimental design and techniques employed, anyway most of the authors agreed that thermal stress set higher energy demands, resulting in high glucose consumption rate and stimulation of glycogenolysis and gluconeogenesis [9,38]. A similar pattern was reported in fish undergoing starvation and crowding stress or captivity [52,53,54]. On the contrary, the intake of diets supplemented with oregano EO led to reduced glucose levels. Several authors associated the reduction in blood glucose to the hypoglycemic effects of carvacrol [55,56,57,58]. By improving insulin sensibility and promoting intracellular glucose uptake, carvacrol treatment may also prevent ROS production and oxidative damage [59]. To some extent, the energy stores depletion during stress exposure [26] could explain the poor growth rates observed with CD compared to D100. On the other hand, the same beneficial effect of EO on growth performances was not observed with D200, likely due to the high EO dose exposure, which appeared to have a deleterious impact [29].

A reduction of triglycerides in fish subjected to temperature shifts was previously reported by other authors [60]. The decrease observed in fish fed on CD was likely due to the depletion at liver level of triglycerides and cholesterol to support the energy cost of gluconeogenesis [53]. On the other hand, the sharp reduction of triglycerides in D100 and D200 was mainly attributable to the hypolipidemic effect of carvacrol rather than to the consumption of triglycerides [61]. The lower cholesterol content in fish fed on experimental diets may be also ascribed to the suppression of 3-hydroxy-3- methylglutaryl coenzyme A reductase (HMG-CoA), a key regulatory enzyme in cholesterol synthesis [62]. Indeed, Kim et al. [63] demonstrated that carvacrol lowers hepatic cholesterol through the downregulation of genes involved in lipogenesis. Our results are in line with the study of Hong et al. [64] which revealed a significant reduction of serum cholesterol when broiler chickens fed on essential oils containing carvacrol as major component.

Serum proteins are key indicator of vital functions, such as humoral defense, coagulation, metabolite transport, and homeostasis [65]. Their levels may be influenced by water quality and seasonal changes or by endogenous factors (e.g., hemodilution and reproductive cycle) [66,67]. In the present study, serum proteins increased according to the temperature rise, reaching the highest peak when exposed to 21 °C. Further switching the temperature up to 25 °C, serum protein decreased, in particular in fish fed on CD. Our results for serum protein showed similarity with the study of Islam et al. [26], which found a decreasing trend in sea bass exposed to thermal stress. In response to the environmental fluctuations, proteins are released into circulation where they are catabolized in order to produce ATP, to support gluconeogenesis and to maintain the physiological homeostasis [68]. The activation of protein catabolism exposes fish to immune dysfunction [69], skeletal muscle degradation [70], growth restriction, and makes fish susceptible to death [71]. On the other hand, the elevated protein levels observed in fish fed on experimental diets may be attributed to EO supplementation, likely due to activation of regulatory and metabolic pathways to protect proteins from degradation. Many studies reported a high protein level and the consequent immuno-stimulating effect in rainbow trout, carp, or catfish treated with carvacrol [72,73,74,75].

Principal component analysis (PCA) based on oxidative stress biomarkers and serum biochemical parameters showed a clear separation of control and experimental groups as a function of temperature changes. However, diet supplementation with 100 ppm of EO seemed to mitigate the effects of high temperatures.

## 5. Conclusions

Summing up, we investigated the physiological responses triggered by temperature changes in European sea bass, and the effectiveness of dietary EO supplementation in counteracting the thermal stress. Under exposure to thermal shift, EO affected fish growth and metabolic biomarkers in a dose-depending manner. The dosage of the EO is crucial to obtain the desired effects and thus deserves to be appropriately investigated. The addition of 100 ppm oregano EO improved growth performances, restored the antioxidant enzyme machinery (SOD and CAT), and activated the non-specific immune system by increasing the serum protein level. On the other hand, a higher dose (200 ppm) of EO was less effective in counteracting the thermal stress and was detrimental to the fish growth. Our findings are crucial for promoting the economic sustainability of dietary supplementation with EO, as low-dose treatments are cheaper than higher dosages. The nutritional strategy we proposed is easily transposable into the field and can benefit different aquaculture sectors, from small-scale fish farmers to intensive productions. The role of EO in regulating the antioxidant enzyme machinery and the non-specific immune system suggests a versatility of application. Likely, EO administration might potentially be effective as preventive treatment and as an alternative to antibiotics in several cultured species, and as sustainable treatment for diseases and stress management in farms of high-income countries. Further investigations should be taken on target tissues to understand biological mechanisms ameliorating fish conditions under temperature changes.

## Figures and Tables

**Table 1:** Nutritional composition of commercial feed ^1^.

Ingredients	Composition (%)
Crude protein	42.00
Crude fat	18.00
Crude fiber	3.20
Ash	9.00
Total carbohydrates	18.80
Mineral supplement	1.40
Vitamin C (mg/Kg)	160.00
Vitamin E (mg/Kg)	160.00
Gross energy (mj/Kg)	18.44

^1^ The feed was supplied by Veronesi Mangimi A.I.A. S.p.A-Italy.

**Table 2:** Chemical composition of *Origanum vulgare* L. essential oil ^1^.

Compounds	Concentrations
Carvacrol Hydrocarbons	60–80% 15%
Citral Beta-caryophyllene	2.5–8.0% 0.5%
Geraniol	0.2%
Limonene	0.3%
Linalool	0.3%
Eugenol	0.1%
Arsenic	<1 mg/kg
Lead	<1 mg/kg
Mercury	<1 mg/kg
Cadmium	<1 mg/kg
Total heavy metals	<10 mg/kg

^1^ The essential oil of *Origanum vulgare* L. was obtained by Farmalabor S.R.L.-Italy.

**Table 3:** Growth performances of sea bass fed on control diet (CD) and experimental diets with different concentration of oregano essential oil: 100 ppm (D100) and 200 ppm (D200)

Growth Parameters	CD	D100	D200	*p*-Value
Initial body weight (g)	12.48 ± 0.70	12.48 ± 0.70	12.48 ± 0.70	n.s.
Final body weight (g)	125.75 ± 2.91 ^b^	142.52 ± 2.11 ^a^	108.82 ± 2.16 ^c^	<0.001
Specific growth rate (%/d)	1.51 ± 0.03 ^b^	1.60 ± 0.04 ^a^	1.41 ± 0.04 ^c^	<0.001

Values are reported as means ± standard deviations. Values followed by different letters (^a^^–^^c^) in the same row are significantly different by Tukey post hoc tests (*p* < 0.05). *p*-values from one-way analysis are also provided. n.s. = not significant.

## Data Availability

Not applicable.

## Supplementary Materials

The following are available online at https://www.mdpi.com/article/10.3390/ani11040982/s1, Table S1: Results of two-way ANOVA evaluating the effects of temperature and feeding treatments on weight values and serum SOD and CAT activities and TBARS, proteins, triglycerides, cholesterol and glucose levels; Table S2: Weight values of sea bass fed on control and experimental diets and exposed to different temperatures.
