# Supplementary material for: A Multi-Biomarker Approach in European Sea Bass Exposed to Dynamic Temperature Changes under Dietary Supplementation with Origanum vulgare Essential Oil
Source: Animals (Basel). 2021 Apr 1;11(4):982. doi: 10.3390/ani11040982 (PMC8066705; doi:10.3390/ani11040982)
Supplement: Supplementary file 1 [file animals-11-00982-s001.zip › Supplementary files/Table S2.docx]

**Table S2**. Weight values of sea bass fed on control diet (CD) and experimental diets with different concentration of oregano essential oil: 100 ppm (D100) and 200 ppm (D200) and, exposed to different water temperature (13, 15, 18, 21, 23 and 25 °C).

|  | **Weight (g)** | | |
| --- | --- | --- | --- |
| **Temperature (°C)** | **CD** | **D100** | **D200** |
| 13 | 12.48 ± 0.70F | 12.48 ± 0.70F | 12.48 ± 0.70F |
| 15 | 22.83 ± 0.82bE | 24.53 ± 0.99aE | 21.19 ± 0.95cE |
| 18 | 38.07 ± 1.14bD | 41.72 ± 1.00aD | 32.57 ± 1.67cD |
| 21 | 59.35 ± 1.55bC | 65.21 ± 1.97aC | 51.71 ± 1.41cC |
| 23 | 87.15 ± 1.35bB | 96.82 ± 1.45aB | 76.94 ± 2.27cB |
| 25 | 125.75 ± 2.91bA | 142.52 ± 2.11aA | 108.82 ± 2.16cA |

Data are reported as means ± standard deviations. Data were submitted to a two-way analysis of variance (ANOVA) and Tukey post hoc tests was used to compare means, with signiﬁcance level of 5%. Different capital letters (A-F) indicate significant differences (*p* < 0.05) among water temperature within the same diet. Different lowercase letters (a-c) indicate significant differences (*p* < 0.05) among diets within the same water temperature.
