# Supplementary material for: A Multi-Biomarker Approach in European Sea Bass Exposed to Dynamic Temperature Changes under Dietary Supplementation with Origanum vulgare Essential Oil
Source: Animals (Basel). 2021 Apr 1;11(4):982. doi: 10.3390/ani11040982 (PMC8066705; doi:10.3390/ani11040982)
Supplement: Supplementary file 1 [file animals-11-00982-s001.zip › Supplementary files/tabella S1.docx]

**Table S1.** Results of two-way ANOVA evaluating the effects of temperature (13, 15, 18, 21, 23 and 25 °C) and feeding treatments (fish fed on control diet and experimental diets with different concentration of oregano essential oil: 100 ppm and 200 ppm) on fish weight, serum SOD and CAT activities, and TBARS, proteins, triglycerides, cholesterol and glucose levels.

| **Parameters** | ***df*** | **F** | ***p*** |
| --- | --- | --- | --- |
| **Weight** | | | |
| Temperature | 4 | 21337.00 | < 0.001 |
| Feeding treatments | 2 | 1339.20 | < 0.001 |
| Temperature $\times$ Feeding treatments | 8 | 143.40 | < 0.001 |
|  | | | |
| **TBARS** | | | |
| Temperature | 4 | 88.65 | < 0.001 |
| Feeding treatments | 2 | 278.86 | < 0.001 |
| Temperature $\times$ Feeding treatments | 8 | 55.01 | < 0.001 |
|  |  |  |  |
| **SOD** | | | |
| Temperature | 4 | 234.50 | < 0.001 |
| Feeding treatments | 2 | 1356.80 | < 0.001 |
| Temperature $\times$ Feeding treatments | 8 | 315.00 | < 0.001 |
|  |  |  |  |
| **CAT** | | | |
| Temperature | 4 | 21.00 | < 0.001 |
| Feeding treatments | 2 | 45.91 | < 0.001 |
| Temperature $\times$ Feeding treatments | 8 | 12.48 | < 0.001 |
|  |  |  |  |
| **Proteins** | | | |
| Temperature | 4 | 67.75 | < 0.001 |
| Feeding treatments | 2 | 73.64 | < 0.001 |
| Temperature $\times$ Feeding treatments | 8 | 3.33 | 0.003 |
|  |  |  |  |
| **Triglycerides** | | | |
| Temperature | 4 | 32964.00 | < 0.001 |
| Feeding treatments | 2 | 6345.00 | < 0.001 |
| Temperature $\times$ Feeding treatments | 8 | 1075.00 | < 0.001 |
|  |  |  |  |
| **Cholesterol** | | | |
| Temperature | 4 | 775.50 | < 0.001 |
| Feeding treatments | 2 | 2298.30 | < 0.001 |
| Temperature $\times$ Feeding treatments | 8 | 210.10 | < 0.001 |
|  |  |  |  |
| **Glucose** | | | |
| Temperature | 4 | 437.20 | < 0.001 |
| Feeding treatments | 2 | 251.10 | < 0.001 |
| Temperature $\times$ Feeding treatments | 8 | 177.70 | < 0.001 |

*df*: degree of freedom;

F: Fisher statistic value;

*p*: *p*-value.
